# Endovascular repair in type A aortic dissection: Anatomical candidacy for currently manufactured stent grafts and conceptual valve-carrying devices for an Endo-Bentall procedure

**DOI:** 10.1093/ejcts/ezad085

**Published:** 2023-03-14

**Authors:** Maximilian Kern, Sven R Hauck, Theresa-Marie Dachs, Lukas Haider, Marie-Elisabeth Stelzmüller, Marek Ehrlich, Christian Loewe, Martin A Funovics

**Affiliations:** Division of Cardiovascular and Interventional Radiology, Department of Bio-medical Imaging and Image-Guided Therapy, Medical University of Vienna, Vienna, Austria; Division of Cardiovascular and Interventional Radiology, Department of Bio-medical Imaging and Image-Guided Therapy, Medical University of Vienna, Vienna, Austria; Division of Cardiovascular and Interventional Radiology, Department of Bio-medical Imaging and Image-Guided Therapy, Medical University of Vienna, Vienna, Austria; Division of Cardiovascular and Interventional Radiology, Department of Bio-medical Imaging and Image-Guided Therapy, Medical University of Vienna, Vienna, Austria; Department of Cardiac Surgery, Medical University of Vienna, Vienna, Austria; Department of Cardiac Surgery, Medical University of Vienna, Vienna, Austria; Division of Cardiovascular and Interventional Radiology, Department of Bio-medical Imaging and Image-Guided Therapy, Medical University of Vienna, Vienna, Austria; Division of Cardiovascular and Interventional Radiology, Department of Bio-medical Imaging and Image-Guided Therapy, Medical University of Vienna, Vienna, Austria

**Keywords:** Endovascular aortic repair, Type A dissection, Valve-carrying conduit

## Abstract

**OBJECTIVES:**

Endovascular treatment has been suggested as an alternative for open surgery for type A aortic dissection, but current devices have severe anatomical limitations. This study assesses the computed tomography-based anatomical suitability of currently manufactured stent grafts as well as 2 embodiments of valve-carrying devices.

**METHODS:**

In a retrospective single-centre cohort of 200 consecutive ascending/arch operations between 2009 and 2018, a total of 112 patients with type A aortic dissections were identified and evaluated for endovascular candidacy based on the locations of the entries, the landing zone diameters/lengths and the supra-aortic vessel origins according to the anatomical instructions for use criteria of 6 commercially available (tubular, branched or fenestrated) stent grafts. Two suggested valve-carrying devices with inner branches or fenestrations for the coronary arteries and branches for the supra-aortic vessels were also evaluated.

**RESULTS:**

The anatomical feasibility for commercial stent grafts ranged from 4% to 21%. The main limitations were proximal landing zone diameter (considering oversizing <15%), length due to dilatation and an entry too close to the sinotubular junction. For the valve-carrying conduits, anatomical feasibility was between 31% and 80%, with the main limiting factors being the diameter of the aortic annulus and its distance to the coronary arteries.

**CONCLUSIONS:**

The anatomical applicability of currently manufactured stent grafts for the treatment of type A aortic dissection is limited mainly by the absence of a suitable proximal landing zone in the ascending aorta and might substantially be improved by anchoring in the aortic annulus using a valve-carrying device that uses either fenestrations or branches for the coronary arteries.

**Figure:**
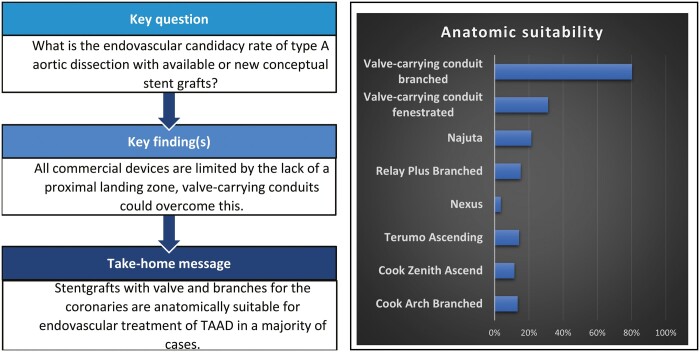

## INTRODUCTION

Open surgery is standard treatment for all ascending aortic pathologies [1]. However, even with recent improvements such as hypothermic circulatory arrest or antegrade and retrograde cerebral perfusion, the procedure is still associated with stagnant mortality rates between 5% and 24% with specific subgroups suffering from even a higher number of deaths [2–6]. Consequently, major centres report a turndown rate of up to 8% of patients deemed unfit for open surgery [7]. Whereas some of these patients may not be eligible for any treatment due to neurological events, a subgroup of these patients might benefit from less invasive treatment options.

Over the last decade, endovascular treatment with dedicated tubular fenestrated or branched stent grafts has been suggested for various pathologies in the aortic arch [8–10]. However, reports of stent graft treatment in the ascending aorta, namely in cases of type A aortic dissection (TAAD), remain anecdotal, mainly due to the lack of a suitable proximal landing zone (LZ) [11]. Recently, valve-carrying stent grafts have been suggested, and a successful first-in-man implant of a stent graft with a valve and branches to engage the coronary arteries has been reported [12–14].

With the currently ongoing development of such stent grafts, anatomical feasibility studies of patients with TAAD regarding the applicability of the currently manufactured and proposed stent grafts are desirable. Not only are such reviews important to address the limitations of currently available devices, they are also needed to direct further stent graft design. To date, however, such investigations are currently limited to single existing devices and show consecutively low suitability rates, and only a single study has evaluated 1 type of a valve-carrying device [15–17].

The present study retrospectively evaluates endovascular candidacies for all currently manufactured ascending and arch stent grafts as well as 2 types of fenestrated and branched valve-carrying devices in a cohort of patients with acute TAAD based on preoperative computed tomography data sets.

## METHODS

### Ethics statement

This retrospective single-centre cohort study was approved by the ethics committee of the Medical University of Vienna (EK 1786/2021). The need for patient-informed consent was waived due to the retrospective nature of the study.

### Patients

All patients who underwent surgery of the ascending aorta to treat a TAAD between 2009 and 2018 and who had adequate preoperative imaging [electrocardiogram (ECG)-gated or short-exposure contrast-enhanced computed tomography angiography (CTA) of the thoracic aorta] were evaluated for this study.

### Image analysis

The appropriate technique to display the proximal sections of the aorta include ECG-gated or short- exposure contrast-enhanced CTA. Such techniques are obligatory to avoid motion artefacts due to the pulsatile activity of the heart. Although the ECG-gated CTA depends on rhythmic cardiac activity and a low heart rate, short-exposure CTA can be performed independently of those parameters and was the method of choice to diagnose an acute aortic syndrome for the majority of patients. The software used to evaluate the scans was syngo.via by Siemens Healthineers, Munich, Germany.

All measurements were made on multiplanar reformatting and 3-dimensional reconstructions via a volume rendering technique in CTA with a slice thickness below 1.5 mm:

Lengths were measured on the outer curvature parallel to the vascular centreline; diameters were measured in the plane perpendicular to the vascular centreline.

Diameters and the distances of the following landmarks were measured: sinotubular junction (STJ), brachiocephalic trunk (BCT), left common carotid artery, left subclavian artery, aortic annulus and proximal coronary artery.

Moreover, the diameters and lengths of potential LZ of the supra-aortic vessels and the widest ascending aortic diameters were determined. Those diameters were measured at the ostium as well as 1, 2 and 3 cm distally. In the case of an elliptical vascular cross-section, the geometrical mean of the maximum and minimum diameters was recorded. In case of dissection at the potential sealing zone, the actual diameter including both lumina was measured.

Furthermore, the angles in which the SAVs originated from the aorta were assessed in a parasagittal plane parallel to the aortic centreline and were recorded. Additionally, the angle and radius of the aortic arch were noted. The radius was measured from the circle that fit in the steepest curve of the inner curvature of the aortic arch; the angle of the aortic arch was defined as the angle of the aortic centreline at the vertex of the steepest curve.

The proximal aortic LZ was defined as the zone between the proximal entry and the STJ. In the case of a valve-carrying conduit (VCC), the LZ was extended to the aortic annulus. The entry was defined as the most proximal disruption of the dissection membrane, and its location was set in relation to the STJ and the BCT. In cases where no entry could be identified, the distal end of the LZ was defined as the proximal end of the dissection membrane. A visualization with further explication of the measurements can be seen in Fig. 2.

### Statistical analyses

The Jarque-Bera test was used to test for normality of data. Normally distributed values are expressed as a mean with a standard deviation; non-normally distributed values are expressed as a median with the interquartile range. Measures of frequency are described as proportion and percentage. Categorial variables are presented as proportions.

The statistical software used was SPSS 27.0 (IBM, Armonk, NY, USA).

### Devices

Anatomical suitability was determined by application of the anatomical criteria provided by the manufacturers (Table 1) of the following commercially available, custom-made (CMD) or off-the-shelf (OTS) tubular, fenestrated and branched stent grafts (Fig. 3) as well as for 2 embodiments of VCC with fenestrations or branches for the coronary arteries as described in Fig. 4: Terumo ascending (Terumo Aortic, Sunrise, FL, USA) (OTS); Cook Zenith Ascend (Cook, Bloomington, Indiana, USA) (OTS); Cook Double Branched (Cook) (CMD); Relay Plus Double Branched (Terumo Aortic) (CMD); Najuta Stent Graft System (Kawasumi Laboratories, Tokyo, Japan) (CMD); Nexus Aortic Arch Stent Graft System (Endospan, Herzliya, Israel) (OTS).

**Table 1.** Stent-graft anatomical requirements by manufacturer

Stent graft/Criteria	Feasibility
Cook Arch Branched feasibility (available dm 24-46 mm)	
−STJ–BCT distance ≥50 mm	112/112 (100%)
−Proximal landing zone ≥20 mm (30 if native aorta)	58/112 (52%)
−Proximal landing zone dm 28-38 mm	40/112 (36%)
−Distal landing zone ≥20 mm	112/112 (100%)
−Landing zone in BCT dm ≤20 mm	74/112 (66%)
−Landing zone in LCCA dm ≤13 mm	111/112 (99%)
−Landing zone BCT ≥20 mm	110/112 (98%)
−Landing zone LCCA ≥20 mm	111/112 (99%)
−Aortic arch radius ≥20 mm	110/112 (98%)
−Aortic arch localized angulation >45°	112/112 (100%)
Anatomical feasibility	15/112 (13%)
Anatomical feasibility w/o proximal LZ	68/112 (61%)
Najuta feasibility (available dm 24-42 mm)	
−Proximal landing zone >20 mm	56/112 (50%)
−Proximal landing zone dm 20-38 mm	41/112 (37%)
−Distal landing zone >20 mm	112/112 (100%)
−Distal landing zone dm 20-38 mm	108/112 (96%)
Anatomical feasibility	24/112 (21%)
Anatomical feasibility w/o proximal LZ	108/112 (96%)
Relay Plus Double Branch feasibility (available dm 22-46 mm)	
−Proximal landing zone ≥30 mm	53/112 (47%)
−Proximal landing zone dm 29-43 mm	67/112 (60%)
−Distal landing zone ≥25 mm	112/112 (100%)
−Distal landing zone dm 19-43 mm	111/112 (99%)
−Landing zone BCT ≥25 mm	109/112 (97%)
−Landing zone BCT dm 7-20 mm	74/112 (66%)
−Landing zone LCCA ≥30 mm	111/112 (99%)
−Landing zone in LCCA dm 7-20 mm	91/112 (81%)
−STJ—BCT distance >65 mm	110/112 (98%)
−STJ—BCT distance >85 mm^a^	106/112 (95%)
−Proximal BCT—distal LCCA distance <45 mm	107/112 (96%)
Anatomical feasibility	17/112 (15%)
Anatomical feasibility w/o proximal LZ	51/112 (46%)
Nexus feasibility (available dm 36-43mm)	
−Proximal landing zone ≥30 mm	53/112 (47%)
−Proximal landing zone dm 29-39 mm	41/112 (37%)
−Distal landing zone aortic dm 26-40 mm	89/112 (79%)
−Landing zone BCT dm 11.5-18.5 mm	50/112 (45%)
−STJ–BCT distance >30 mm	112/112 (100%)
−Landing zone descending aorta ≥30 mm	112/112 (100%)
−Landing zone BCT ≥20 mm	110/112 (98%)
−Angle between BCT and aortic arch ≥125°	110/112 (98%)
Anatomical feasibility	4/112 (4%)
Anatomical feasibility w/o proximal LZ	31/112 (28%)
Terumo Ascending feasibility (available dm 22-50 mm)	
−Distance STJ–BCT 60–270	112/112 (100%)
−Proximal landing zone ≥10 mm	58/112 (52%)
−Proximal landing zone dm 22-43 mm	72/112 (64%)
−Distal landing zone ≥10 mm^b^	50/112 (45%)
−Distal landing zone dm 22-43 mm	89/112 (79%)
Anatomical feasibility	16/112 (14%)
Anatomical feasibility w/o proximal LZ	40/112 (36%)
Cook Zenith Ascend feasibility (available dm 24-46 mm)	
−Proximal landing zone ≥20 mm	58/112 (52%)
−Proximal landing zone dm 20-42 mm	67/112 (60%)
−Distal landing zone ≥10 mm^b^	50/112 (45%)
−Distal landing zone dm 20-42 mm	79/112 (71%)
Anatomical feasibility	13/112 (12%)
Anatomical feasibility w/o proximal LZ	35/112 (31%)
Valve-carrying conduit with fenestrations feasibility	
−Proximal landing zone ≥10 mm	62/112 (55%)
−Proximal landing zone dm 20-43 mm	72/112 (64%)
−Applicable to attachable stent graft w/o proximal LZ	112/112 (100%)
−Aortic annulus dm ≤30 mm	100/112 (89%)
−Aortic annulus proximal coronary artery distance ≥10 mm	101/112 (90%)
Anatomical feasibility	35/112 (31%)
Valve-carrying conduit with branches feasibility	
−Applicable to attachable stent graft w/o proximal LZ	112/112 (100%)
−Aortic annulus dm ≤30 mm	100/112 (89%)
−Aortic annulus proximal coronary artery distance ≥10 mm	101/112 (90%)
Anatomical feasibility	90/112 (80%)

a If proximal device length is 60 instead of 45 mm.

b +10 mm is safety distance to entry.

BCT: brachiocephalic trunk; dm: diameter;  LCCA: left common carotid artery; LSA: left subclavian artery; LZ: landing zone; OTS: off the shelf; STJ: sinotubular junction.

Oversizing of 10–20% was taken into account compared to the actual vessel diameter of the dissected aorta.

For both embodiments of the VCC, the combination with a branched or fenestrated arch stent graft, the distal LZ was considered suitable if any of the commercial stent grafts would be distally applicable. For the fenestrated VCC, an LZ of 10 mm proximal to the STJ was defined as necessary. For the branched VCC, no such requirements were obligatory because it would only be anchored within the aortic annulus.

Anatomical criteria regarding the aortic valve were extracted from the instructions for use and from previous publications, which translated to a diameter of 30 mm or a circumference of 92 mm [18]. The commercially available valve considered as a model was the transcatheter aortic valve implant (TAVI) Edwards Sapien (Edwards Lifesciences, Irvine, CA, USA). Longitudinally, a minimal distance from the aortic annulus to the proximal coronary artery of 10 mm was required.

## RESULTS

### Patients

**Figure 1:**
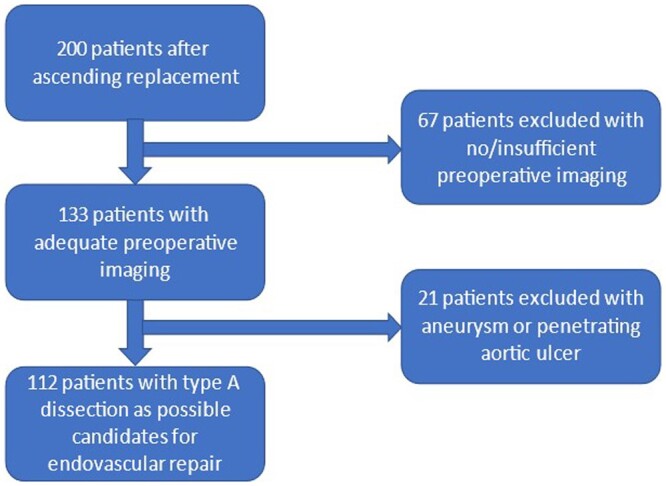
Selected pool of patients.

Out of 200 patients who underwent operations of the ascending aorta or the aortic arch between 2009 and 2018, a total of 133 patients had adequate preoperative images (ECG-gated or short-exposure, contrast-enhanced CTA of the thoracic aorta); of these, 112 patients were operated on for a TAAD and form the basis of this report (Fig. 1).

**Figure 2:**
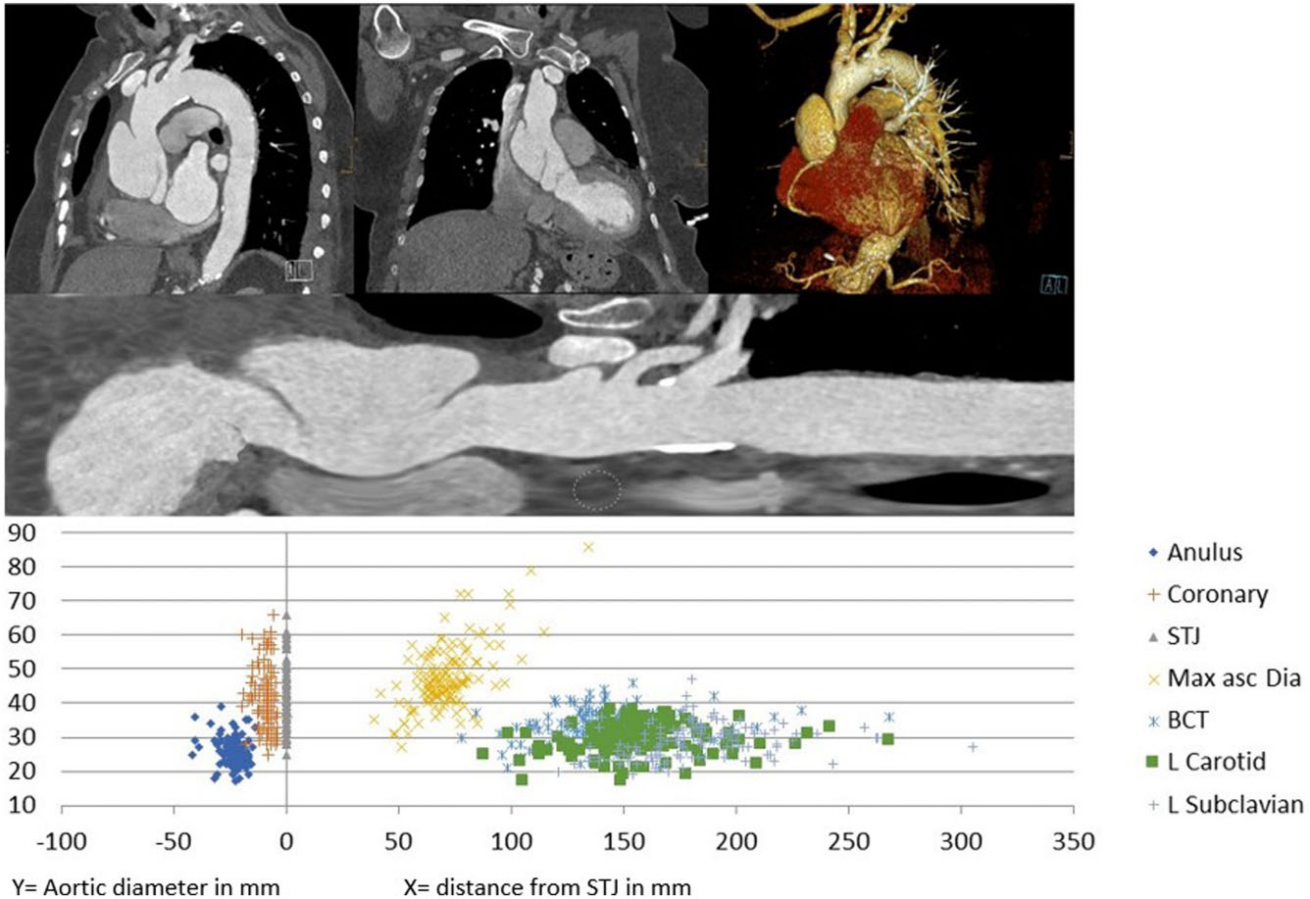
Diameter of the dissected aorta at certain anatomical landmarks in relation to their distance from the sinotubular junction. A negative distance equals a position proximal to the sinotubular junction. Annulus: annulus aortae; BCT: origin of the brachiocephalic trunk; Coronary: more proximal coronary ostium; L Carotid: origin of the left common carotid artery; L Subclavian: origin of the left subclavian artery; Max asc Dia: maximal diameter of the tubular ascending aorta; STJ: sinotubular junction.

**Figure 3:**
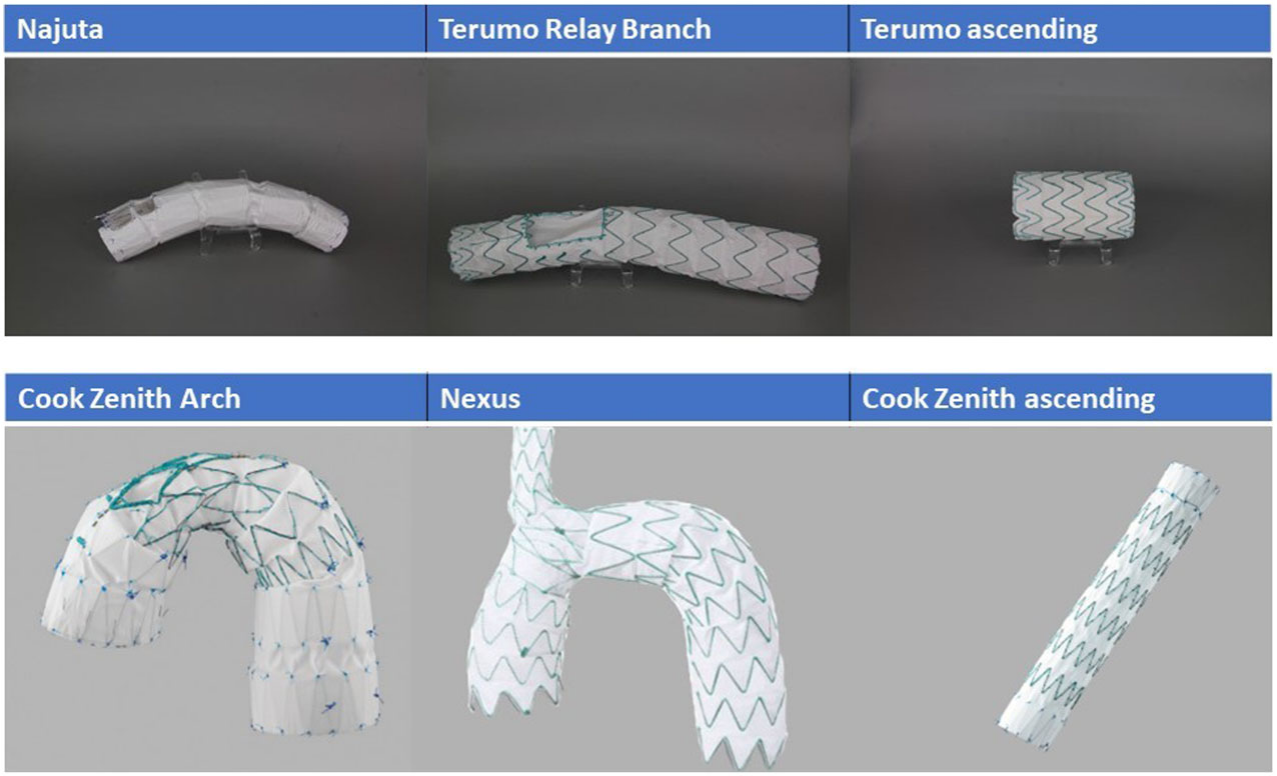
Evaluated commercially available stent grafts.

**Figure 4:**
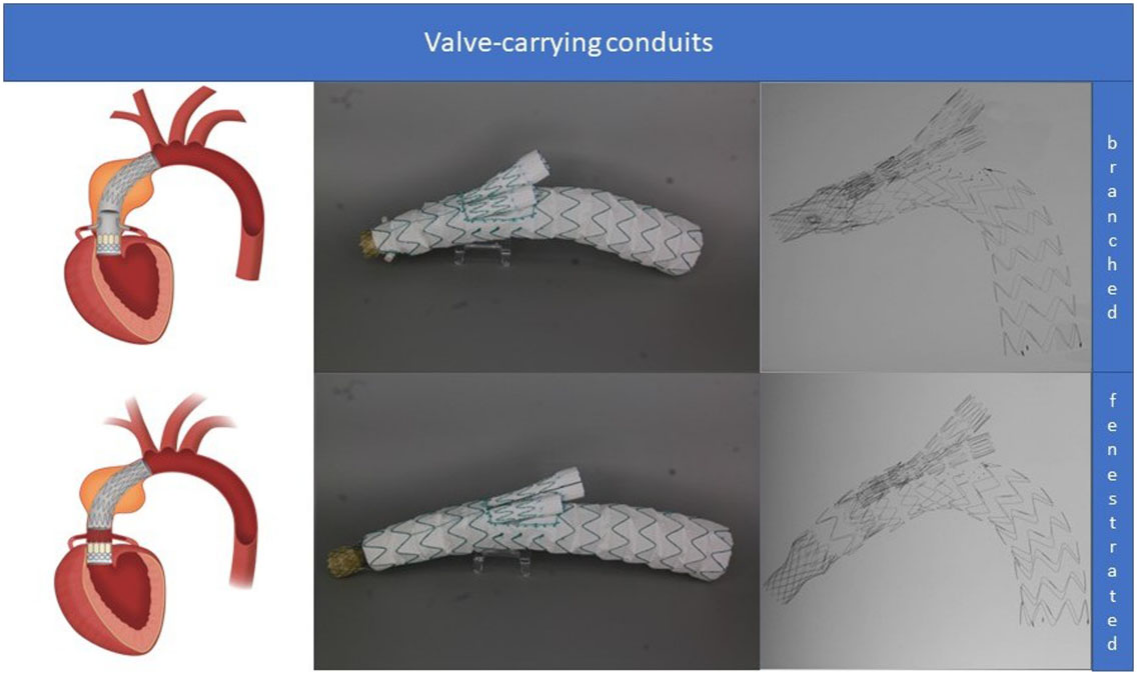
Conceptional valve-carrying conduits.

### Anatomical measurements

The mean anatomical characteristics of the 112 patients were as follows: The aortic diameter at the STJ was 42 mm, 48 mm at zone 0, 32 mm at zone 1 and 24 mm at zone 2. The length of the ascending aorta (STJ–BCT) was 125 mm. The dissection reached the STJ in 102 patients, and a singular entry was visible in 73 patients. The distance from the STJ to the entry was 42 mm. This resulted in an ascending aortic LZ length of 48 mm.

For the TAVI-related measurements, the mean aortic annulus diameter was 26 mm, the distance from the annulus to the proximal coronary artery was 14 mm and the distance from the annulus to the STJ was 24 mm (Table 2).

**Table 2.** Mean anatomical measurements of the aortic arch and access vessels

Measurements	Mean/median in mm or number of cases
Bovine arch	16/112 (14%)
Visible entry	73/112 (65%)
Dissection up to STJ	102/112 (91%)
Zone 0/1 border to entry	67.5 (p25: 30; p75: 90)
STJ diameter	42.29 (SD: 9.08)
Aortic anulus diameter	26.36 (SD: 3.71)
Distance proximal coronary artery–anulus	13.84 (SD: 3.71)
Distance aortic anulus–STJ	23.51 (SD: 5.12)
Ascending aorta diameter	46(p25: 42; p75: 52.5)
Descending aorta diameter	29.5 (p25: 26; p75: 33)
BCT diameter	19 (p25: 16; p75: 22)
RCCA diameter	8 (p25: 7; p75: 9)
LCCA diameter	10,5 (p25: 9; p75: 12)
Dissected LCCA	13/112 (12%)
LSA diameter	14 (p25: 12; p75: 16)
Distance STJ-BCT	121 (p25: 108; p75: 133)
Landing zone ascending aorta	47.80 (SD: 58.01)
Landing zone descending aorta	289 (p25: 261; p75: 308.75)
Landing zone BCT	46.71 (SD: 11.96)
Landing zone RCCA	95 (p25: 80.5; p75: 107)
Landing zone LCCA	126 (p25: 111; p75: 139)
Landing zone LSA	44 (p25: 39; p75: 52)
Length zone 1	14 (p25: 11; p75: 17)
Diameter zone 1	32.34 (SD: 4.86)
Distance proximal BCT—distal LCCA	32.47 (SD: 6.67)
Length zone 2	23 (p25: 20; p75: 27)
Diameter zone 2	29.06 (SD: 4.46)
Distance LCCA-LSA	10 (p25: 6; p75: 1329 (p25: 25; p75: 32.5)
Diameter zone 3	141 (p25: 133.75; p75: 148)
Angle BCT-Aorta (α-angle)	138.40 (SD: 14.52)
Angle LCCA-Aorta (β-angle)	148.50 (SD: 13.83)
Angle LSA-Aorta (ɣ-angle)	105.5 (p25: 95; p75: 115)
Angle aortic arch	33 (p25: 26; p75: 38.5)
Radius aortic arch	49/112 (44%)
Mild aortic calcification	15/112 (14%)
Kinking of any one branch	9 (p25: 7; p75: 10)
Iliac artery diameter	5/112 (4%)
Iliac artery kinking	

BCT: brachiocephalic trunk; LCCA: left common carotid artery; LSA: left subclavian artery; RCCA: right common carotid artery; SD: standard deviation; STJ: sinotubular junction.

### Stent graft applicability

For the branched or fenestrated commercially available stent grafts, anatomical applicability was mainly limited by the proximal LZ diameter, with suitable diameters as follows: Cook branched 36% (40/112), Najuta 37% (41/112), Relay plus 60% (67/112) and Nexus 37% (41/112). The second most limiting factor was the proximal LZ length: Cook branched, 52% (58/112); Najuta, 50% (56/112); Relay plus, 47% (53/112); and Nexus, 47% (53/112). Only for the Nexus stent graft was the BCT LZ diameter a more limiting factor than the proximal LZ with suitability in 45% (50/112) of cases.

For the tubular ascending stent grafts of Terumo and Cook, the length of the distal LZ was the most limiting parameter with 45% (50/112) suitable cases. The proximal LZ was suitable in 52% (58/112) of the cases for both devices.

The importance of the proximal LZ as the main limiting parameter is emphasized by the results of feasibility when all limitations from the proximal LZ are excluded: 61% (68/112) Cook branched; 96% (108/112) Najuta; 46% (51/112) Relay plus; 28% (31/112) Nexus; 36% (40/112) Terumo ascending; and 31% (35/112) Cook Zenith Ascend.

Detailed data about the feasibility of each parameter are given in Table 1. Applying all parameters, the overall anatomical applicability for the respective commercial devices was as follow: 14% (15/112) Cook branched; 21% (24/112) Najuta; 15% (17/112) Relay plus; 4% (4/112) Nexus; 14% (16/112) Terumo ascending; and 12% (13/112) Cook Zenith Ascend. The fenestrated VCC had an overall applicability of 31% (35/112), whereas the branched VCC showed the highest applicability of 80% (90/112).

The fenestrated version of the VCC was less limited by the proximal LZ diameter (63%; 72/112), but rather from the proximal LZ length with 55% (62/112) suitable patients. A tubular version ending proximally to the BCT reached the overall feasibility of 14% (16/112), while the potential combination with tubular, fenestrated or branched TEVAR devices increased feasibility to 31% (35/112).

The branched VCC showed appreciably higher anatomical feasibility, which was only limited by the diameter of the aortic annulus (89%; 100/112) and the required distance from the aortic annulus to the proximal coronary artery (90%; 101/112). The overall feasibility of a tubular device to the BCT was 30% (34/112), whereas a combination with tubular, fenestrated/branched TEVAR devices increased overall feasibility to 80% (90/112).

## DISCUSSION

Our data show that the overall anatomical feasibility of commercially available stent grafts to treat type A dissection is remarkably low, ranging from 4% to 21%. This finding is congruent with the existing literature and is mostly due to the fact that the proximal sealing of the stent graft is compromised [19].

As expected, the LZ diameter was the most limiting parameter because a dissection originating in the ascending aorta is often accompanied with significant dilatation of the vessel up to 32% of its original diameter [20]. Obviously, both manufacturing constraints and the fragility of the dilated aortic wall preclude the application of large-diameter stent grafts in this region. The maximal manufactured diameter for both the commercially available and conceptual stent grafts is 50 mm. Taking into consideration an oversizing of 15%, the maximal treatable aortic diameter is approximately 43 mm. The second limitation of the proximal LZ is its length. In 53% of the patients, the LZ length was below 30 mm and in 49%, it was below 20 mm.

The high proportion of short LZ is only partly attributable to the fact that the entry is often close to the STJ [17, 21]. In our data set, a proportion of entries could not be clearly identified. In these cases, the most proximal point of the dissection represented the end of the LZ. If in such cases the dissection propagates into the aortic bulbus, the LZ can be completely eliminated. Similarly, the same limitation applied for the distal LZ with tubular stent grafts where the entry was not identifiable.

The incorporation of a valve allows us to shift the LZ to the aortic annulus and to supply the coronary arteries through the stent graft.

One concept is the fenestrated VCC: Previous descriptions suggested the perfusion of the coronary arteries through a circular bare metal stent segment that connects an ascending stent graft with a transcatheter aortic valve, which provides a proximal LZ in the aortic annulus [14, 17]. However, to exclude the false lumen of the dissected ascending aorta, a sealing zone at the level of the STJ is still required. In accordance with previous publications, the length of this zone was deemed suitable with 10 mm, but obviously, no experimental or clinical data exist to support this assumption. One might argue that a 10-mm LZ is low compared to requirements for LZ in other locations. On the other hand, we know from TAVI practice that precise deployment within a range of a few millimeters can be routinely performed and that there are encouraging results from ascending stent grafts with high deployment accuracy [8, 22]. In addition, the valve presents a natural anatomical barrier for further proximal propagation of the dissection. Because the stent graft would be connected to the implanted valve, correct engagement of the distal structures would mostly be a matter of preinterventional planning. Although the fenestrated VCC would enhance applicability, it would still be unsatisfyingly low, mainly due to cases with the proximal entry too close to the coronary ostia. Another limitation may be the propagation of the dissection into the coronary or carotid arteries. Although carotid dissection could preclude fenestrated endovascular treatment, the placement of a bridging stent graft into the true lumen during branched TEVAR may prove beneficial if creation of an SINE can be avoided. We did not observe propagation of the dissection into the coronary arteries in this cohort of patients who were transferred to this tertiary centre; this fact could represent a selection bias because patients in this series have survived the computed tomography scan and have undergone open repair.

To overcome the remaining anatomical limitations, the need for wall apposition in the ascending aorta would have to be eliminated completely. This goal may be achieved if the bare metal stent segment is replaced by two retrograde inner branches directly distal to the implanted valve. Complete exclusion of the false lumen could be secured if the coronary arteries are connected via bridging stent grafts from a femoral approach. The anatomical applicability of this concept would depend mainly on a suitable diameter of the aortic annulus. It would also isolate the coronary arteries from potential propagation of the dissection, but it requires that the patency of the coronary stent grafts be maintained. The first in-human implant based on this concept was recently successfully performed [12].

The fenestrated and the branched VCC can both be combined with the distal branched or the fenestrated stent graft segments to cover the aortic arch. For both options, data about safety and durability in dissections are scarce. Currently, branched devices suffer from higher complication rates, namely strokes, whereas fenestrated devices have only recently been approved for dissections [9].

Our results favour a branched VCC combined with a fenestrated arch component, with an applicability rate of up to 80%. Nevertheless, in cases where the dissection propagates into the supra-aortic vessels, caution must be advised: While anatomically suitable per the instructions for use, experience with endovascular treatment in such cases is extremely limited. To avoid propagation of the dissection with limited flow in the distal true lumen on the one hand, and of retrograde filling of the false aortic lumen via the dissected supra-aortic vessel on the other hand, additional interventions may be required [23].

Currently, an acute type A dissection with its high complication and mortality rates may prove to be a too dangerous, complex and fast-paced indication for this new hypothetical approach [24]. Endovascular treatment in TAAD carries elevated interventional risk, namely stroke and rupture, and there is only limited experience using endovascular valve technology in aortic regurgitation. However, especially during the current coronavirus disease pandemic, we have seen a rise in the numbers of patients with chronic type A dissections who suffered from delayed hospitalization [25]. These patients may be candidates if this approach is ever to be tested, because the dissection has already proved to be stable enough to allow for urgent instead of acute intervention. In addition, such interventions need extensive preclinical and animal testing. Although the fenestrated VCC could be tested in swine, a suitable animal model for the branched VCC approach will be difficult to establish, because sheep and swine have ascending aortas of insufficient length and diameter.

The implications of this study have to be regarded in view of its limitations. First, this is a retrospective single-centre study, which limits the quality of the available data in number and representation. For example, some studies reported significantly lower rates of entry detection, which would decrease the discussed stent graft applicability rates [26]. With the more widespread availability of short-exposure CTA, the visualization of entries will probably improve. Second, the applicability rates were evaluated solely upon the instructions for use. Although this is a common approach, in clinical practice the actual applicability may be altered by different factors because operators or manufacturers can change some parameters in custom-made devices or even in the operating room. Moreover, device-unrelated factors such as access vessel feasibility were not analysed. The strength of the study lies in its evaluation of the widest selection of available stent grafts as well as in its discussion of hypothetical devices to overcome current limitations.

## CONCLUSION

Anatomical applicability of commercially available stent grafts for the treatment of TAAD is low. The most limiting factor is the proximal LZ, either because the entry is too close to the STJ or because no entry can be identified, and the dissection involves the STJ. Fenestrated or branched VCCs may overcome this limitation, creating a more proximal LZ because they could be anchored in the aortic annulus. Further investigations are necessary to develop this concept to a testable level.

## Data Availability

Data not contained in the manuscript can be made available upon reasonable request to the corresponding author.

## Abbreviations

BCT: brachiocephalic trunk
CMD: custom-made
CTA: computed tomography angiography
ECG: electrocardiogram
LZ: landing-zone
OTS: off-the-shelf
STJ: sinotubular junction
TAAD: type A aortic dissection
TAVI: trans-catheter aortic valve implant
VCC: valve-carrying conduit
